# Molecular and physiological characterization of the chitin synthase B gene isolated from *Culex pipiens pallens* (Diptera: Culicidae)

**DOI:** 10.1186/s13071-019-3867-z

**Published:** 2019-12-30

**Authors:** Xiaoshan Yang, Qi Yin, Yang Xu, Xixi Li, Yan Sun, Lei Ma, Dan Zhou, Bo Shen

**Affiliations:** Department of Pathogen Biology, Nanjing Medical University, Nanjing, China

**Keywords:** *Culex pipiens pallens*, Insecticide resistance, Chitin synthase, RNA interference

## Abstract

**Background:**

The growth and development of insects is strictly dependent on the precise regulation of chitin synthase (CHS), which is absent in vertebrates and plants. Therefore, CHS represents an attractive target for insecticides. At present, the research on the CHS gene in mosquitoes, especially its biological functions, remains limited.

**Results:**

The full-length cDNA of the chitin synthase B gene in *Culex pipiens pallen*s (*CpCHSB*) was prepared and consists of 5158 nucleotides with an open reading frame (ORF) of 4722 nucleotides encoding a protein of 1573 amino acid residues. Among different tissues, *CpCHSB* gene is mainly expressed in the midgut tissue with the highest expression in adult mosquitoes. Knockdown of *CpCHSB* in the larval stage significantly lowered the chitin content (16.5%) decreased body size (reduced by 25.6% in the larval stage and by 25.6% in the adult stage), and diminished reproduction (20%). Injecting siCHSB into adult mosquito mainly decreased reproduction (27%).

**Conclusions:**

*CpCHSB* plays essential roles in growth and development, by severely reducing larval chitin content, midgut permeability, and reducing the number of female mosquito offspring. These results indicate that *CHSB* may serve as a potential novel target for exploring biosafe insecticides.

## Background

Mosquitoes are among the most dangerous vectors for many severe diseases including malaria, dengue fever, Zika fever, yellow fever and chikungunya [1–3]. These mosquito-borne diseases cause serious public health problems and large economic losses in tropical and subtropical areas. At present, mosquito control programmes mainly rely on the application of chemical insecticides, but their widespread and improper use leads to the development of resistance in vectors. Of the 80 countries in which malaria is endemic that provided data for 2010–2017, 68 have reported resistance to at least one class of insecticide, and 57 of those reported resistance to two or more classes [1]. In addition, since chemical insecticides accumulate in the environment, they may have adverse effects on non-target organisms, including humans [4]. New and more accurately targeted tools are indeed needed [5–7].

Chitin, a linear polymer of β-(1,4)-*N*-acetyl-d-glucosamine (GlcNAc), is the second most abundant natural polysaccharide after cellulose [8]. It is widely distributed in arthropods, nematodes and fungi, but is absent in vertebrates and plants. In insects, chitin is the key structural component of the cuticular exoskeleton, trachea, head capsule, cuticular lining of the foregut, hindgut, and peritrophic membrane (PM) that lines the lumen of the midgut [9]. Chitin functions as a scaffold material and plays an important role in protecting insects against external invasion and the abrasion of food [10]. Therefore, chitin may be a potential selective target for novel insect control strategies.

Chitin synthase (CHS), an enzyme that transfers NDP-acetylglucosamine (NAP-GlcNAc) to polymerise chitin [10], catalyses the last step of chitin synthesis. During development and metamorphosis, the chitin content in insects fluctuates as a complex function of the activities of CHS. In general, insect CHS fall into two different classes; *CHSA* and *CHSB*. Although both share some conserved motifs (DXD [hD(S/A)DT], EDR[E(D562E)R], and QRRRW[(Q601N)RR(R604L)(W605Y)]) [6, 11] and have some basic properties in common, they are believed to have different functions during insect growth and development. *CHSA* is responsible for chitin synthesis in cuticle and trachea in *Anopheles gambiae* [12], *Tribolium castaneum* [13] and *Locusta migratoria* [14], whereas *CHSB* is mainly located in the midgut and is presumably responsible for synthesising chitin in the PM during the feeding stage in *Anopheles gambiae* [12], *Ostrinia nubilalis* [10] and *L. migratoria* [14]. Moreover, a recent study showed that both CHSA and CHSB proteins were present in the newly formed compound eyes of *Anopheles gambiae* pupae according to immunohistochemical analysis [12].

Previous functional analyses of the *CHSB* gene using RNA interference (RNAi) in several different insect species demonstrated its requirement for various physiological processes. In *Bactrocera dorsalis*, CHSB regulates the chitin content of the midgut, and thereby affects growth and development. CHSB in *Bombyx mori* can affect the chitin synthesis-dependent form of the PM and is related to food intake and the molting process. Silencing of the *CHSB* gene in *Leptinotarsa decemlineata* significantly reduces food consumption, decreases chitin content and retards larval growth. Although the *CHSB* gene has been characterised in several mosquito species [12, 15], physiological functional information is rather limited. In *Aedes aegypti*, mature and immature larvae treated with dsRNA targeting *CHSB* gene catalytic sites displayed decreased viability, and a few larval and adult survivors displayed altered morphology, including a reduction in chitinous bristles located on the abdominal segments and thorax [4]. In addition, relatively high *CHSB* gene expression occurs in the *A. gambiae* pupal stage compared with larval stages [12]. This finding suggests that although CHSB may not be useful for PM-associated chitin development in pupae these enzymes may have other physiological roles, suggesting that further study is needed to understand the functions of the enzyme encoded by *CHSB*.

*Culex pipiens pallens* is widely distributed in China [16]. This species is the major vector of the Japanese encephalitis virus as well as *Wuchereria bancrofti*, the causative agent of filariasis [17]. In the present study, we performed molecular characterization and expression profiling of the *CHSB* gene in *Cx*. *pipiens pallen*s (*CpCHSB*) and explored the biological functions by gene silencing *via* microinjection experiments.

## Methods

### Mosquitoes

*Culex pipiens pallens* larvae were reared at 28 ± 1 °C in water supplemented with rat chow. Adult mosquitoes were reared at 27 °C and 60–70% relative humidity with unlimited access to water and 7% (w/v) sucrose solution under a 16:8 h light:dark photoperiod. To allow females to lay eggs, 3-day-old females were fed fresh mouse blood [18, 19].

### *CpCHSB cDNA* cloning and sequencing

Total RNA was isolated from 3-day-old female mosquitoes, 10 mosquitoes as a biological replicate using RNAiso Plus reagent (TaKaRa, Tokyo, Japan) according to the manufacturer’s instructions. The *CpCHSB* cDNA sequence was determined by sequencing six overlapping PCR fragments (Additional file 1: Table S1), and primers were designed based on the ORF of *Culex quinquefasciatus* (GenBank: XM_001846240.1). The 5′- and 3′-RACE ends were amplified using a SMARTer RACE 5′/3′ Kit following the instructions of the manufacturer (TaKaRa). PCR was carried out with rTaq polymerase (TaKaRa) in a 50 μl reaction mixture containing 2.5 μl cDNA templates, 1 μl GSP (10 μM), 25 μl 2× SeqAmp buffer, 1.0 μl SeqAmp DNA Polymerase, 5 μl 10× UPM and 15.5 μl ddH_2_O. Thermal cycling involved 20 cycles of denaturation at 95 °C for 30 s, annealing at 55–65 °C (based on the primer annealing temperature) for 30 s, and extension at 72 °C for 2–3 min (based on the size of the expected fragment). Amplified PCR products were analyzed on a 1% agarose gel and purified using a Gel Extraction Mini Kit (Tiangen, Beijing, China). The product was placed at −20 °C. Purified DNA was ligated into the pMD18-T vector and sequenced completely from both directions.

### Nucleotide and amino acid sequence analysis

The amino acid sequence of *CpCHSB* was translated using the ExPASy website (https://web.expasy.org/translate/). Similar sequences were searched using the non-redundant protein sequence (nr) database of the National Center for Biotechnology Information (NCBI) website (http://blast.ncbi.nlm.nih.gov/Blast.cgi). Prediction of molecular weight, isoelectric point and transmembrane helices was conducted using (http://cn.expasy.org/tools/pi_tool.html), and N-glycosylation sites were also analyzed (http://www.cbs.dtu.dk/services/NetNGlyc/) [20, 21].

### Analysis of developmental stage and tissue-specific expression of *CpCHSB* by real-time quantitative PCR (RT-qPCR)

To assess developmental expression of *CpCHSB*, total RNA was extracted from eggs (0 and 24 h), fourth-instar larvae, pupae (0 and 24 h) and adult females (0, 24, 48 and 72 h post-eclosion and 24, and 48 h post-blood meal). Total RNA was isolated from 5 adult female mosquitoes for each biological replicate using RNAiso Plus reagent (TaKaRa). For tissue-specific expression analysis of *CpCHSB*, RNA was extracted from six different tissues (head, foregut, midgut, hindgut, Malpighian tubules and carcass) from fourth-instar larvae [8, 10]. Total RNA was isolated from 10 fourth-instar larvae for each biological replicate using RNAiso Plus reagent (TaKaRa). RT-qPCR was performed using a LightCycler® 96 Instrument (Roche, Switzerland, Germany) with Power SYBR Green PCR Master Mix (Applied Biosystems, Vancouver, USA), according to the manufacturer’s protocol. The reaction volume (10 μl) contained Power SYBR Green PCR Master Mix, specific forward and reverse primers (Additional file 2: Table S2), and diluted cDNA. The relative expression levels were normalized *β-actin* as an internal control [22] using the 2^−ΔΔCq^ method [23]. The quality of RNA was assessed by gel electrophoresis and spectrophotometry. The integrity of RNA was by analysed by visualising bands for *28S* and *18S* ribosomal RNA [24]. All experiments were performed in triplicate and included three biological replicates and at least four technical replicates. Data are presented as the mean transcription ratio ± standard error of the mean (SE).

### Analysis of morphological characteristics

Small interfering RNA for silencing the *CpCHSB* gene (siCHSB) and negative control (NC) RNA was purchased from Gene Pharma (Shanghai, China; Additional file 2: Table S2). Approximately 0.30 μg of siCHSB or NC was injected into the abdomen of third-instar larvae [25] and the thorax of 1-day-old adult female mosquitoes [19], and the same volume of wild type (WT) and negative control (NC) were injected as controls. After 72 h of recovery, knockdown levels of the *CpCHSB* transcript were measured by RT-qPCR.

After injecting siCHSB, NC into third-instar larvae, the body length of 4-day-old final-instar larvae was measured between the initial vertex of the head capsule and terminal vertex of abdominal segments. Adult body length was estimated from wing length, measured from the apical notch to the axillary margin [23]. Lengths were measured using Fiji Image J software [26]. To minimise measurement errors, all appendage measurements were determined by a single researcher, and data from at least four replicate experiments were combined for statistical comparisons. All experiments included three biological replicates and at least four technical replicates. Significant differences were analyzed using Student’s t-tests.

### Chitin analysis

The chitin content of larvae and adult mosquito was quantified as described previously by Arakane et al. [27]. After injection of siRNA, extract from control and siRNA larvae or adults of a similar weight were subjected to absorbance measurement at 585 nm using a microplate reader. Standard curves were prepared from stocks of 0.05 to 2.25 mM GlcNAc. The same weight of mosquito was used for normalisation of chitin content across different samples [28, 29].

This method can quickly assess whether the larval midgut is defective. Larvae were dissected after 72 h injected with siCHSB, NC and midgut tissue was transferred to a glass vial. Samples were incubated with Rhodamine B (final concentration 1 mM in water; Solarbio, Beijing, China) on a shaker for 1 min, rinsed thoroughly with water, and stained midgut was photographed using a Coolpix 4300 (Nikon, Tokyo, Japan) [30].

Third-instar larvae were injected with siRNA, and after 72 h samples were collected for section staining. The head and tail were removed, and the body including the middle part of the intestine was incubated in fixing solution (4% paraformaldehyde) overnight. After paraffin embedding, samples were dewaxed and stained with Fluorescent Brightener 28 (Sigma-Aldrich, Shanghai, China) for 10 s, washed three times with water for 1 min each time, counterstained with propidium iodide for 10 s, washed three times with water for 1 min each time, and excess water was removed from the air-dried slide. Anti-fluorescent stain was added dropwise for sealing and samples were observed under a fluorescence microscope [31].

### Statistical analysis

SPSS 23.0 and GraphPad Prism 6.0 software [31] was used for statistical analysis. Differences in efficiency, length, and chitin content between treatments (WT, NC, siCHSB) were compared using Student’s t-tests [31]. To compare more than two sets, we used one-way analysis of variance analysis (ANOVA) followed by Tukey’s *post-hoc* tests. All statistical values are presented as the mean ± standard error of the mean (SE). All experiments were performed using at least three independent cohorts, and *P-*values of < 0.05 were considered statistically significant.

## Results

### *CpCHSB* cDNA and deduced amino acid sequences

The full-length *CpCHSB* cDNA was amplified by multiplex PCR using a cDNA template from mosquitoes, and RACE-PCR was used to amplify the 5′ and 3′ regions. The full-length cDNA of *CpCHSB* (GenBank: MK303758) spans 5158 nucleotides and includes an open reading frame (ORF) of 4722 nucleotides encoding a protein of 1573 amino acid residues. The *CpCHSB* cDNA includes a 5′-untranslated region (UTR) 335 nucleotides upstream of the initiation codon (ATG) and a 101 nucleotide 3′ UTR, ending with a poly(A) tail. The putative CpCHSB protein contains three domains; an N-terminal domain with seven transmembrane helices, a highly conserved central domain, and a C-terminal domain with an additional seven transmembrane helices. Sequences characteristic of chitin synthase (QRRRW and EDR) were identified in CpCHSB.

**Fig. 1:**
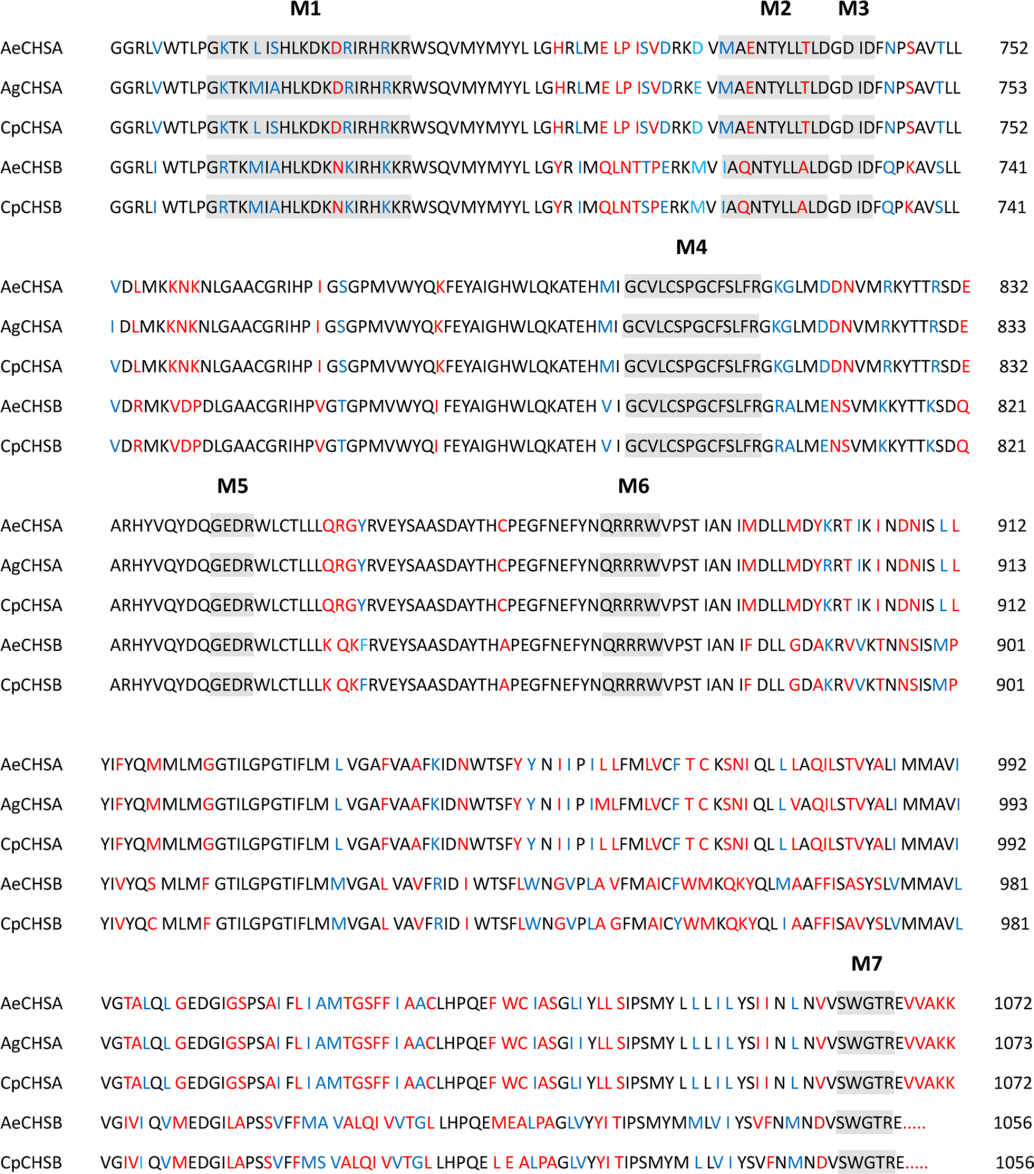
Alignment of the conserved catalytic domain of chitin synthases from three mosquito species Seven characteristic motifs (M1–M7) in insect chitin synthases are highlighted. Dashes are used to denote gaps introduced to maximise alignment. *Abbreviations*: Ae, *Aedes aegypti*; Ag, *Anopheles gambiae*; Cp, *Culex pipiens pallens*

We also derived amino acid sequences of CHSA and CHSB, both containing seven highly conserved motifs (M1–M7; Fig. 1). Since these motifs are present in all chitin synthases, they are considered characteristic sequences [6].

### Tissue-specific expression of *CpCHSB*

**Fig. 2:**
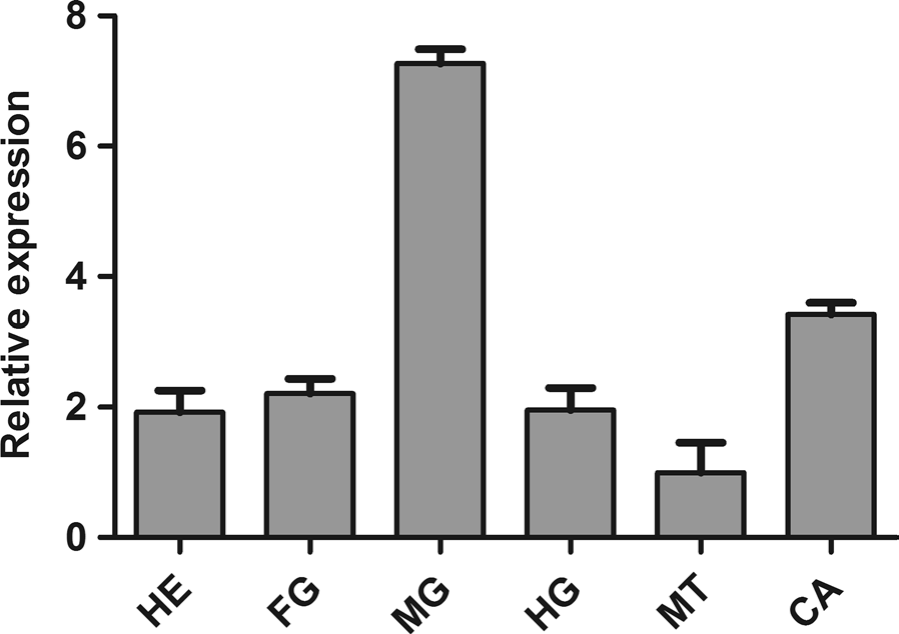
Expression profiles of *CpCHSB* in different tissues of fourth-instar *C. pipiens pallens* larvae. Tissues include head (HE), foregut (FG), midgut (MG), hindgut (HG), Malpighian tubules (MT) and carcass (CA). Relative expression levels were calculated based on the lowest expression value, which was ascribed an arbitrary value of 1. Results are shown as the mean ± SE

Our results showed that *CpCHSB* was expressed in the head, foregut, midgut, hindgut, Malpighian tubules and carcass of fourth-instar larvae (Fig. 2), with highest levels in the midgut and lowest levels in Malpighian tubules.

### Expression of *CpCHSB* during different developmental stages

**Fig. 3:**
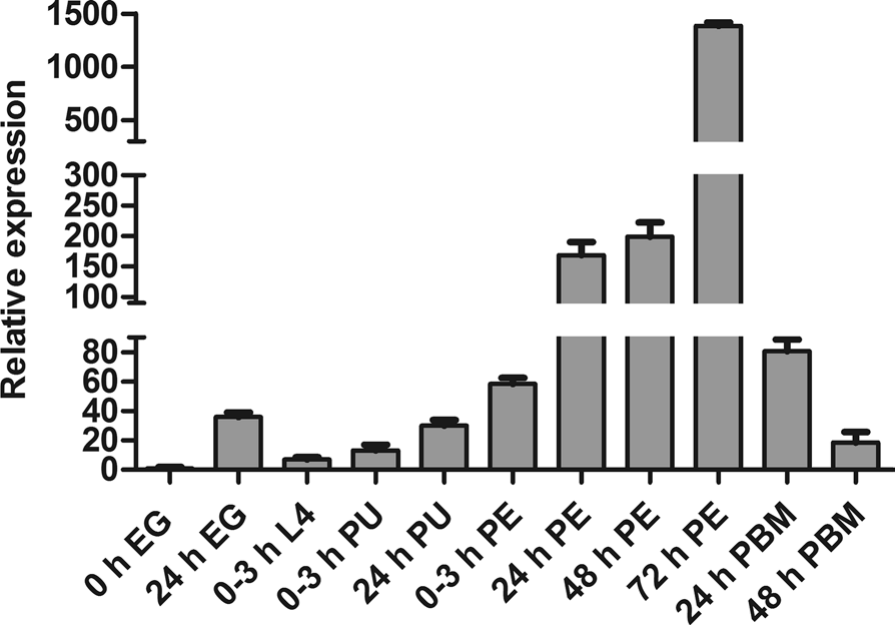
Expression profiles of *CpCHSB* during different developmental stages. Stages include egg (EG), fourth-instar larvae (L4), pupae (PU), post-eclosion (PE), and post-blood meal (PBM). Relative expression levels were calculated based on the lowest expression value, which was ascribed an arbitrary value of 1. Results are shown as the mean ± SE

Expression of *CpCHSB* during different developmental stages from eggs to adults was examined by RT-qPCR (Fig. 3). The results showed that *CpCHSB* was expressed at all stages, and expression gradually increased from larvae and peaked in adults after 72 h of eclosion. Relative *CpCHSB* expression levels in eggs, larvae and pupae were significantly lower than in adults.

### RNAi silencing of *CpCHSB* affects growth

**Fig. 4:**
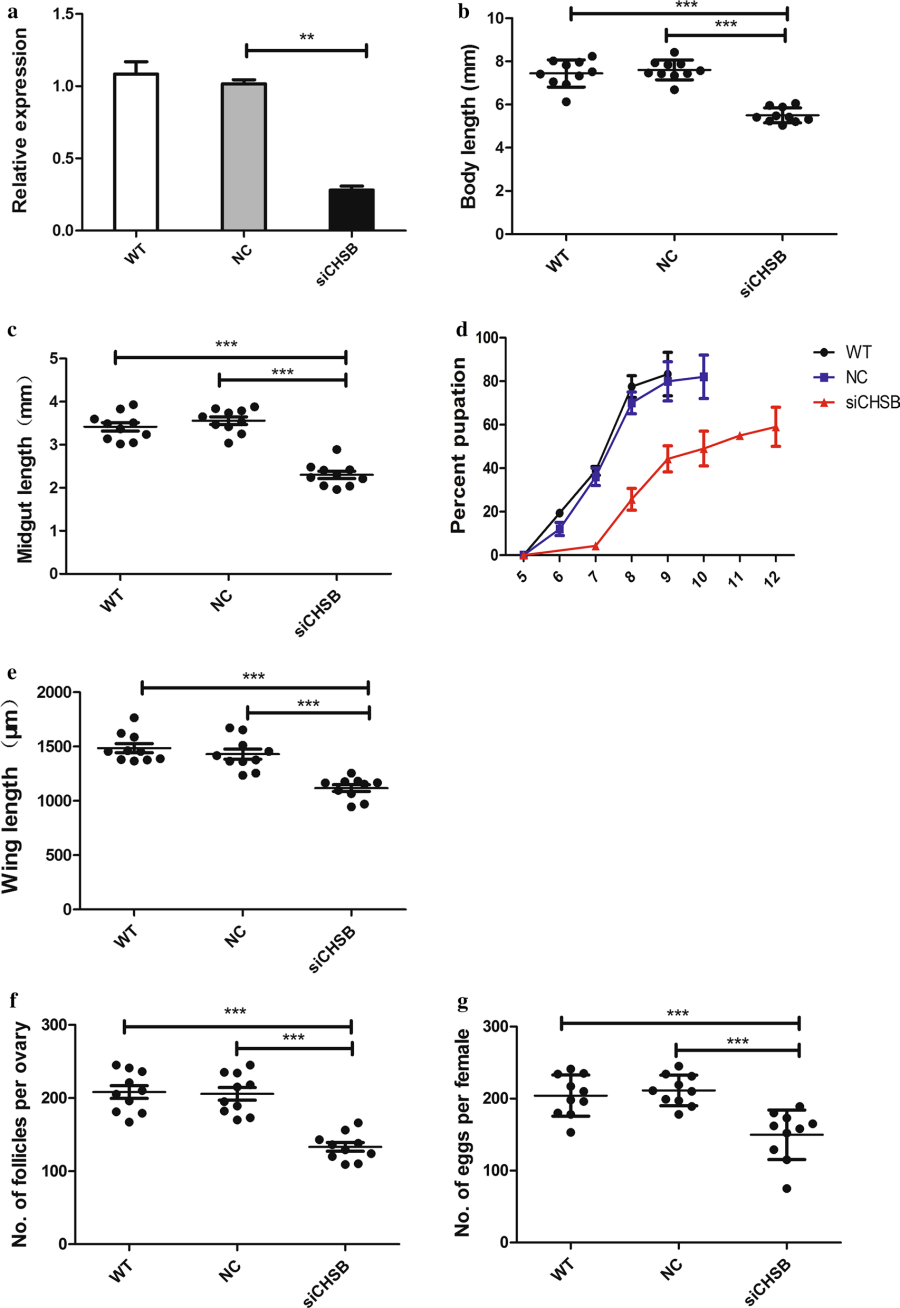
RNA interference (RNAi) of *CpCHSB* in third-instar larvae (*n* = 200). **a** Expression levels of *CpCHSB* at 72 h after injecting siCHSB assessed by RT-qPCR. **b** siCHSB injection into third-instar reduces body length in fourth-instar larvae, as well as midgut length (**c**). **d** Percentage of pupation (x-axis) after egg-hatching. **e** Comparison of wing length in wild type (WT), negative control (NC) and siCHSB adults. **f** Number of follicles per ovary and the number of eggs per female mosquito (**g**) after injecting (*n* = 50) siCHSB. All surviving individuals were used for measurements, and results are shown as the mean ± SE (Student’s t-tests: **P < 0.01, ***P < 0.001)

To evaluate the function of *CpCHSB* in mosquitoes, we injected siCHSB into 200 third-instar larvae to knock down expression of *CpCHSB*, and 30% died during the course of growth due to the injection, while 10% died before the fourth-instar stage, 7% were unable to enter the pupal stage and died before pupation, and 13% pupae not forming in mosquitoes and died before eclosion. Levels of *CpCHSB* transcription were decreased (by 65%, Student’s *t*-tests: *t*_(4)_ *= *5.429, *P* = 0.003) at 72 h after injection with siCHSB (Fig. 4a). Compared with the NC group, the siCHSB group displayed smaller larval body size (reduced by 25.6%, *t*_(18)_ = 10.925, *P* < 0.0001) [23] (Fig. 4b), smaller midgut size (reduced by 40%, *t*_(18)_ = 11.414, *P* < 0.0001) (Fig. 4c), and delayed and reduced pupation (Fig. 4d). Wing length is considered a feature associated with and used to assess body size in mosquitoes [32]. Using this estimate, adult mosquitoes in the siCHSB group also displayed smaller body size (reduced by 25%, *t*_(18)_ = 11.566, *P* < 0.0001) (Fig. 4e). Continued observation showed that the ovarian volume at 72 h post-blood meal (PBM) was smaller in the siCHSB group, there were fewer follicles present (reduced by 24.5%, *t*_(18)_ = 5.132, *P* < 0.0001) (Fig. 4f), and after deposition the number of eggs was also decreased compared with the control group (reduced by 25.6%, *t*_(18)_ = 4.534, *P* < 0.0001) (Fig. 4g, Additional file 3: Figure S1).

### RNAi silencing of *CpCHSB* affects reproduction in adult females

**Fig. 5:**
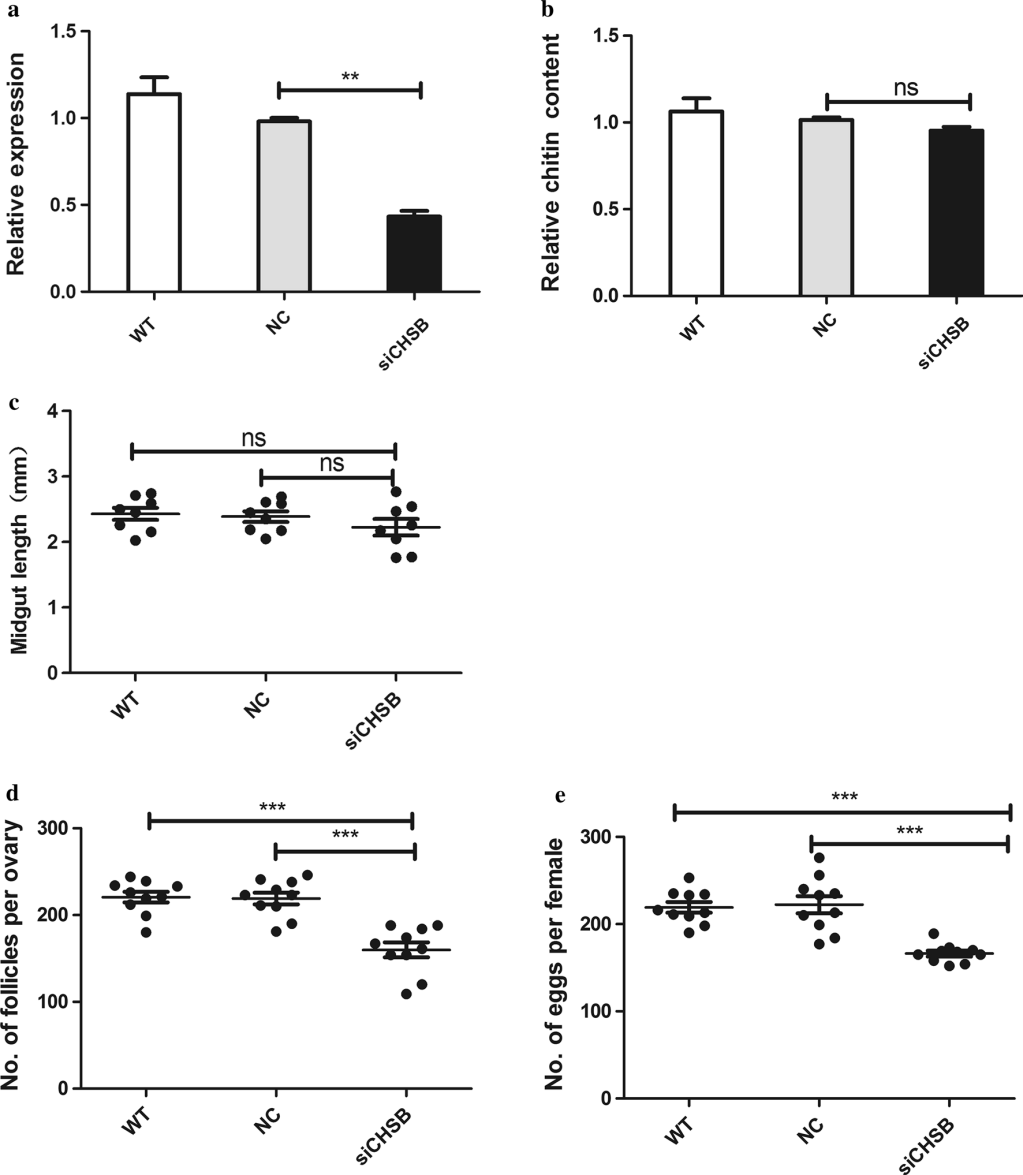
*CpCHSB* gene suppression by RNAi at 1 day after injection (*n* = 200) in adult mosquitoes. **a** Expression levels of *CpCHSB* at 72 h after injecting siCHSB assessed by RT-qPCR. The group injected with siCHSB show a reduction in *CpCHSB* expression of 53% compared with the control group. **b** Relative chitin content at 72 h after siCHSB injection. **c** Midgut length at 72 h after siCHSB injection. **d** Number of follicles per ovary and number of eggs per female mosquito (**e**) after injecting siCHSB. Results are shown as the mean ± SE (Student’s t-tests; **P < 0.01, ***P < 0.001, ns, not significant)

A total of 200 1-day-old female mosquitoes were injected with siCHSB, and the RNAi efficiency was confirmed by RT-qPCR. *CpCHSB* transcription levels were decreased (by 58%, Student’s t-tests: *t*_(4)_ *= *5.424, *P* = 0.006) after siCHSB injection (Fig. 5a), but knockdown of *CpCHSB* expression did not alter the body size, length of the midgut, or chitin content. However, we evaluated ovarian development in female mosquitoes at 72 h PBM, and found that the ovary was reduced in volume, the number of follicles had declined (by 26.4%, *t*_(18)_ = 5.414, *P* < 0.0001) (Fig. 5d), and fewer eggs were deposited (reduced by 27%, *t*_(18)_ = 4.482, *P* < 0.0001) in the siCHSB group compared with the NC group (Fig. 5e, Additional file 4: Figure S2).

### RNAi silencing of *CpCHSB* affects chitin content in the midgut

**Fig. 6:**
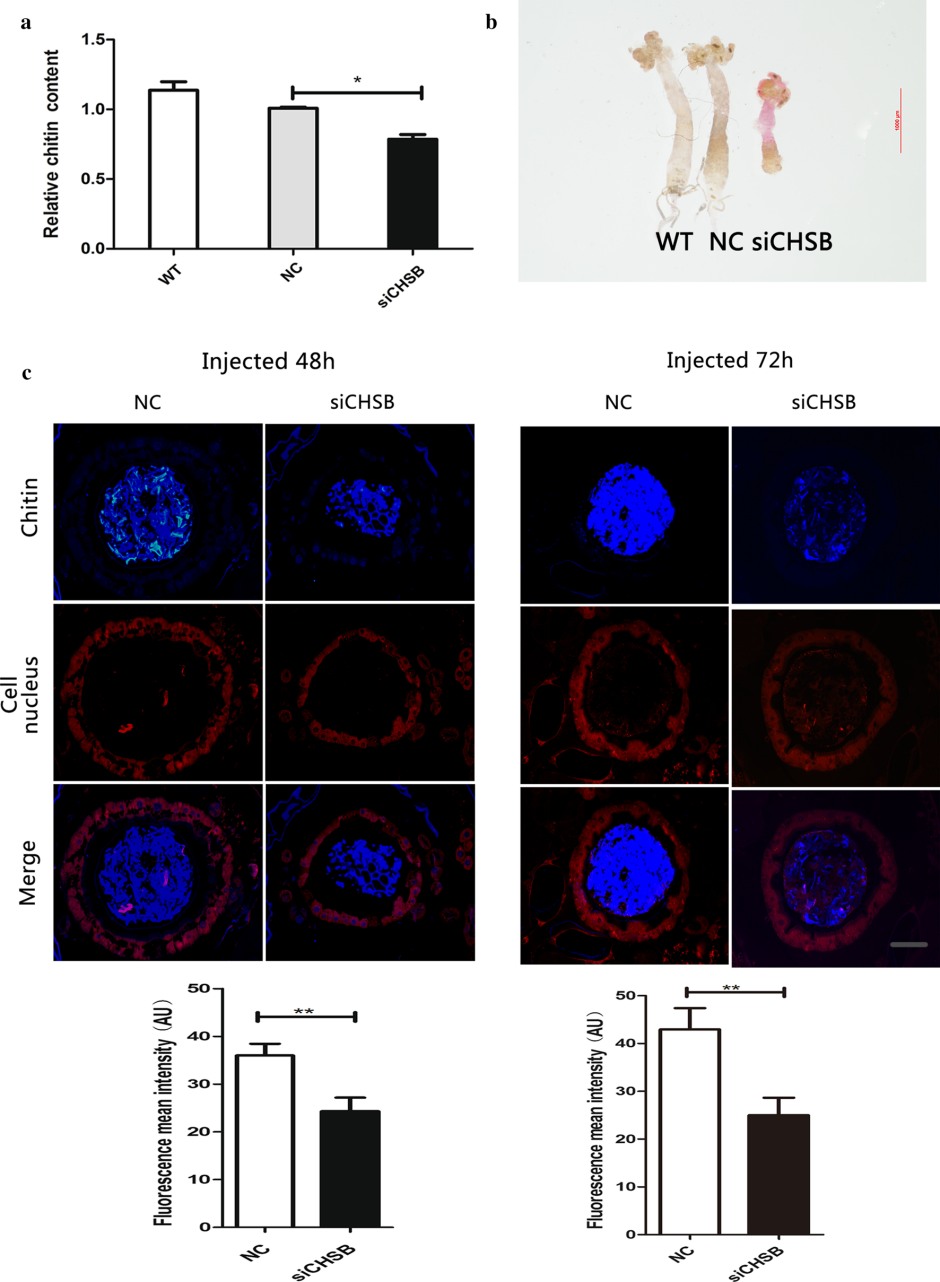
Effect of *CpCHSB* silencing in third instar larvae on chitin content. **a** Relative chitin content in fourth-instar larvae at 72 h after siCHSB injection (*n* = 10). **b** Rhodamine B staining of the midgut of fourth-instar larvae isolated after 72 h after siCHSB injection. **c** Chitin staining in the midgut at 48 and 72 h after siCHSB injection (*n* = 10). Results are shown as the mean ± SE (Student’s t-tests; *P < 0.05, **P < 0.01). *Scale-bar*: 50 μm

At 72 h after siCHSB injection into 200 third-instar larvae, the chitin content of fourth-instar larvae were decreased (by 28%, Student’s t-tests: *t*_(18)_ = 2.7317, *P* = 0.021) (Fig. 6a). To determine whether knockdown of *CpCHSB* affects the permeability of the larval midgut, we employed the chemical marker Rhodamine B to stain the midgut, and the midgut was more translucent in the siCHSB group (Fig. 6b). We also performed eosin staining and chitin staining in the midgut by injecting at 48 h and 72 h, respectively, and observed a significant decrease in chitin content in the midgut of the siCHSB group (Fig. 6c) [33].

## Discussion

In the present study, we report the first full-length cDNA encoding *CHSB* from *C. pipiens pallens*. We conducted bioinformatics, catalytic domain and phylogenetic analyses, and all confirmed its status as a *CHSB* gene. Isolation of the *CHSB* cDNA allowed us to study the physiological functions of this gene *in vivo*.

CHSB is mainly responsible for synthesis of chitin in the midgut. The primary functions of the midgut include digestive enzyme production and nutrient absorption of digestion products [34]. Herein, *CpCHSB* was mainly expressed in the midgut, and knockdown during the larval stage resulted in shorter midgut size. Moreover, the chitin content of the midgut was lower in the siCHSB group, which likely had a weakened epidermis structure. These results indicate an impaired midgut function after knockdown of *CpCHSB*. It is well known that energy available during larval growth has a strong influence on the growth and development of mosquitoes [23]. In our experiment, both larval and adult body size were significantly smaller in siCHSB groups than in the NC group. Also, pupation was delayed in the siCHSB group. Therefore, we hypothesized that knocking out *CpCHSB* may reduce the length of the midgut and the accumulation of chitin in the midgut, which affects the intake of nutrients by mosquitoes, leading to the failure of normal growth and development. Mosquitoes must ingest a blood meal to obtain the nutrients required for oogenesis [35]. We therefore evaluated ovarian development in siCHSB-injected females at 72 h PBM and the ovary volume was smaller, the number of follicles was decreased, and egg deposition was diminished. Our results suggest that knocking down the expression of *CpCHSB* during the larval stage not only affects the growth and development of mosquitoes, but also has a serious impact on offspring.

We discovered that knockdown of *CpCHSB* in 1-day-old adults did not cause any changes in body size, the length of the midgut, or the chitin content. However, in the siCHSB group the volume of the ovary was decreased, there were fewer follicles, and egg deposition was diminished. Therefore, although the external morphology of mosquitoes was not altered significantly, knockdown had a serious impact on reproduction, indicating that *CpCHSB* plays an important role in reproduction in adult mosquitoes.

We also found that knocking down the expression of *CpCHSB* during the larval stage resulted in a lower rate of egg deposition compared with knockdown in adults, indicating that silencing of *CpCHSB* had a less pronounced effect in adult mosquitoes. The above results indicate that even though *CHSB* could be used as a target gene for insecticides in future, the duration of application may have a pronounced influence on the effects. Our results suggest that controlling the expression of *CHSB* in larvae may be more effective for mosquito vector control.

*CpCHSB* was found to be expressed during all developmental stages, indicating a role throughout the life-cycle. *CpCHSB* expression was highest in adults, consistent with results in *B. dorsalis* [11] and *A. gambiae* [12]. Highest expression was observed in the midgut, but *CpCHSB* was also expressed in many other tissues, suggesting that targeting of this gene may be more extensive than first thought. We suspect that *CHSB* is not only functional in the midgut, and this warrants further exploration.

Chitin is an attractive target for insecticides because the chitin synthesis pathway does not exist in vertebrates and plants. However, resistance and non-target toxicity affected their use in vector control [36]. Recently, a large number of benzoylurea derivatives have been synthesised, and 15 benzoylurea chitin synthesis inhibitors have been commercialized [37]. However, benzoylurea derivatives have detrimental effects on some beneficial species, such as bees, aquatic organisms and natural predators, when administered by dispersion, which led to a ban on flufenoxuron in the European Union in 2011 [38]. Our present results indicate that CHSB could be a new target for chitin synthesis inhibitors. Whether CHSB could be applied to the field mosquito control remains to be further investigated.

## Conclusions

We obtained the full-length cDNA encoding *CpCHSB* from *C. pipiens pallens*, which was mainly expressed in the midgut, and was more highly expressed in adult mosquitoes than in larvae. We found that knockdown of *CpCHSB* at different stages produced different effects. Knockdown in the larval stage resulted in shorter body length, shorter midgut length, decreased chitin content, delayed pupation, decreased survival rare, and shorter adult body length in the siCHSB group compared with the NC group. Knockdown in the adult stage did not alter body length, midgut length, or chitin content in the siCHSB group, but reduced the number of ovarian follicles, and decreased egg production. Our results revealed that *CpCHSB* was essential for growth and development, which sheds new light on the characteristics and functions of CHSB in *C. pipiens pallens.* This finding could lead to the development of future vector control applications.

## Supplementary information

**Additional file 1: Table S1.** PCR primers used to amplify the full-length *CpCHSB* cDNA from *Culex pipiens pallens.*

**Additional file 2: Table S2.** Primers used for RT-qPCR analysis and siRNA silencing of *CpCHSB.*

**Additional file 3: Figure S1.** Morphological variations in WT, NC and siCHSB with RNA interference (RNAi) in third-instar larvae.

**Additional file 4: Figure S2.** Morphological variations in WT, NC and siCHSB by RNAi at day 1 after injection in adult mosquitoes.

## Data Availability

Data supporting the conclusions of this article are included within the article and its additional files. Raw data are available from the corresponding author upon request. The sequence was submitted to the GenBank database under the accession number XM_001846240.1.

## Abbreviations

CHS: chitin synthase
RACE: rapid amplification of cDNA ends
GSP: gene-specific primers
UPM: universal primer mix
NCBI: National Center for Biotechnology Information
CHSA: chitin synthase A
CHSB: chitin synthase B
CpCHSB: chitin synthase B gene in *Culex pipiens pallens*
siCHSB: small interfering RNA for silencing the *CpCHSB* gene
ORF: open reading frame
GlcNAc: β-(1,4)-*N*-acetyl-d-glucosamine
PM: peritrophic membrane
NAP-GlcNAc: NDP-acetylglucosamine
PBM: post-blood meal
NC: negative control
SE: standard error of the mean
WT: wild type
UTR: untranslated region
