# Supplementary material for: Molecular and physiological characterization of the chitin synthase B gene isolated from Culex pipiens pallens (Diptera: Culicidae)
Source: Parasit Vectors. 2019 Dec 30;12:614. doi: 10.1186/s13071-019-3867-z (PMC6937787; doi:10.1186/s13071-019-3867-z)
Supplement: Supplementary file 1 — Additional file 1: Table S1. PCR primers used to amplify the full-length CpCHSB cDNA from Culex pipiens pallens. [file 13071_2019_3867_MOESM1_ESM.docx]

| \| **Additional file 1: Table S1.** PCR primers used to amplify the full-length *CpCHSB* cDNA from *Culex pipiens pallens* \| \| \| \| \| --- \| --- \| --- \| --- \| \| **DNA fragment** \| **Primer name** \| **Primer sequence (5′→3′)** \| **PCR product size (bp)** \| \| 1 \| CpCHSB-F1 \| GAGTCCACCAGGACAACAAAC \| 1014 \| \| CpCHSB-R1 \| TGCTGAAGATGTAGCACAGATG \|  \| \| 2 \| CpCHSB-F2 \| GAGATGGAGGCAATCCTGA \| 1103 \| \| CpCHSB-R2 \| AGCAGGTAGTACATGTACATCACCT \|  \| \| 3 \| CpCHSB-F3 \| CTCAACTCGTACGTCAAGATTCT \| 1048 \| \| CpCHSB-R3 \| GTGAGTATACAGCCGAGATGAAG \|  \| \| 4 \| CpCHSB-F4 \| CACGATCTTCCTCATGATGG \| 870 \| \| CpCHSB-R4 \| AGGACTACTGGGAATGACAGAGT \|  \| \| 5 \| CpCHSB-F5 \| GTCCATACCGGACTATCTGTTCT \| 265 \| \| CpCHSB-R5 \| CAGATCTTCCGTCTTGACGTC \|  \| \| 6 \| CpCHSB-F6 \| GTCCAAACGAGTCAAGCAAA \| 317 \| \| CpCHSB-R6 \| CGTCTTCGTCCTCTGTATCTTC \|  \| \| 7 \| 5′RACE-GSP \| GATTACGCCAAGCTTGGATTCGTTTACGGAACGGGACAGCAGTC \| 935 \| \| 8 \| 3′RACE-GSP \| GATTACGCCAAGCTTCTCCGTCGCTGGACTGTTCAGCTGTCT \| 1532 \| |  |  |  |
| --- | --- | --- | --- | --- | --- | --- | --- | --- | --- | --- | --- | --- | --- | --- | --- | --- | --- | --- | --- | --- | --- | --- | --- | --- | --- | --- | --- | --- | --- | --- | --- | --- | --- | --- | --- | --- | --- | --- | --- | --- | --- | --- | --- | --- | --- | --- | --- | --- | --- | --- | --- | --- | --- | --- | --- | --- | --- | --- | --- | --- | --- |

*F=forward; R=reverse. *GSP= gene-specific primer.
