# Supplementary material for: Molecular and physiological characterization of the chitin synthase B gene isolated from Culex pipiens pallens (Diptera: Culicidae)
Source: Parasit Vectors. 2019 Dec 30;12:614. doi: 10.1186/s13071-019-3867-z (PMC6937787; doi:10.1186/s13071-019-3867-z)
Supplement: Supplementary file 2 — Additional file 2: Table S2. Primers used for RT-qPCR analysis and siRNA silencing of CpCHSB. [file 13071_2019_3867_MOESM2_ESM.docx]

| **Additional file 2: Table S2.** Primers used for RT-qPCR analysis and siRNA synthesis of *CpCHSB* | | | |
| --- | --- | --- | --- |
| **Application of primers** | **Primer name** | **Primer sequence (5′→3′)** | **PCR product size (bp)** |
| RT-qPCR | CpCHSB-F | GTGTGAATGTCATCATTCCAGATG | 214 |
|  | CpCHSB-R | CATCGAGAAGCTCTGGATGTG |  |
|  | B-actin-F | AGCGTGAACTGACGGCTCTTG | 140 |
|  | B-actin-R | ACTCGTCGTACTCCTGCTTGG |  |
| siRNA synthesis^a^ | siCHSB-F | GGUCUCGUGUACUACAUAATT |  |
|  | siCHSB-R | UUAUGUAGUACACGAGACCTT |  |
|  | NC-F | GCGACGAUCUGCCUAAGAUDTDT |  |
|  | NC-R | AUCUUAGGCAGAUCGUCGCDTDT |  |

*F=forward; R=reverse. ^a^siRNA primer F and R are complementary, without product.
